# Proteína C-reativa como Marcador Prognóstico de Mortalidade no Primeiro Ano após Implante de Válvula Aórtica Transcateter em Estenose Aórtica

**DOI:** 10.36660/abc.20190715

**Published:** 2021-11-01

**Affiliations:** 1 Universidade Federal Fluminense Hospital Antonio Pedro – Cardiologia Niterói RJ Brasil Universidade Federal Fluminense - Hospital Antonio Pedro – Cardiologia, Niterói, RJ – Brasil; 2 Hospital Pró-Cardíaco – Hemodinâmica Rio de Janeiro RJ Brasil Hospital Pró-Cardíaco – Hemodinâmica, Rio de Janeiro, RJ – Brasil

**Keywords:** Proteina C- Reativa, Inflamação, Biomarcadores, Implante de Prótese de Valva Cardíaca, Substituição da Valva Aórtica Transcateter, Prognóstico, Estenose Aórtica

## Abstract

**Fundamento:**

A proteína C-reativa (PCR) é um biomarcador de inflamação preditor de eventos adversos em procedimentos cardiovasculares. Na avaliação do implante da válvula aórtica transcateter (*transcatheter aortic valve implantation*, TAVI) em relação ao prognóstico de longo prazo ainda é incipiente.

**Objetivo:**

Avaliar a PCR como marcador prognóstico no primeiro ano pós-TAVI na estenose aórtica (EAo).

**Métodos:**

A PCR foi avaliada na primeira semana do peroperatório numa coorte de casos retrospectiva com EAo. Correlacionou-se a PCR pré- e pós-TAVI com a mortalidade e foram pesquisados fatores preditores de mortalidade em 1 ano. Realizada regressão de Cox multivariada para identificar os preditores independentes de óbito em 1 ano.

**Resultados:**

Estudados 130 pacientes submetidos a TAVI, com mediana de idade de 83 anos, sendo 49% deles do sexo feminino. A PCR pré-TAVI elevada (> 0,5 mg/dL) ocorreu em 34,5% dos casos. O pico de PCR foi 7,0 (5,3-12,1) mg/dL no quarto dia. A mortalidade em 1 ano foi 14,5% (n = 19), sendo maior nos grupos com PCR pré-TAVI elevada (68,8% vs 29,1%; p = 0,004) e pico de PCR ≥ 10,0 mg/dL (64,7% vs 30,8%; p = 0,009). Os fatores preditores independentes de mortalidade foram insuficiência renal aguda (IRA) [razão de risco (RR) = 7,43; intervalo de confiança de 95% (IC95%), 2,1-24,7; p = 0,001], PCR pré-TAVI elevada [RR = 4,15; IC95%, 1,3-12,9; p=0,01] e hemotransfusão volumosa [HR = 4,68; 1,3-16,7; p = 0,02].

**Conclusões:**

A PCR pré-TAVI elevada mostrou-se fator preditor independente de mortalidade no primeiro ano, assim como a ocorrência de IRA e hemotransfusões volumosas.

## Introdução

A estenose aórtica (EAo) fibrocálcica é uma doença degenerativa que, segundo projeções, deve ter sua casuística triplicada no Brasil nos próximos 20 anos, devido ao envelhecimento da população.^1^

O implante da válvula aórtica transcateter (*transcatheter aortic valve implantation*, TAVI) é um tratamento em crescente utilização entre idosos, um grupo em que ocorre a inflamação sistêmica crônica de baixa intensidade (*inflammaging*),^2^ cuja presença está associada a maior: (1) disfunção de órgãos e fragilidade; (2) comprometimento do sistema imunológico e risco de infecções; e (3) taxa de eventos cardiovasculares (CV) e mortalidade.^3^ Essa inflamação sistêmica soma-se à inflamação valvar aórtica no processo degenerativo valvar desde o estágio inicial de infiltração lipídica^4^ até a fase tardia de calcificação e neovascularização dos folhetos.^5^ Sendo assim, tanto a inflamação sistêmica quanto a valvar encontram-se presente antes da realização do TAVI e aumentam em graus diferentes após a sua realização, conforme as técnicas e estratégias adotadas.

Entretanto, há um pequeno número de estudos sobre o papel da inflamação sistêmica relacionada ao prognóstico no médio e longo prazos pós-TAVI através dos biomarcadores. O presente estudo avaliou os níveis de inflamação sistêmica antes e durante 1 semana após a realização do TAVI através da dosagem de proteína C reativa (PCR) sérica e correlacionou essa dosagem com o prognóstico em 1 ano.

## Métodos

### População

Este é um estudo observacional, tipo coorte, retrospectivo, que incluiu pacientes sintomáticos com EAo grave que se submeteram a TAVI em um hospital privado entre junho de 2009 e maio de 2015. Nesse período, foram realizados 137 procedimetnos de TAVI em válvula nativa, sendo excluídos quatro casos de complicações mecânicas do procedimento com óbito em 24 horas, e três casos realizados em pacientes em estado crítico. Foram estudados 130 pacientes portadores de EAo grave, com sintomas de insuficiência cardíaca (IC), angina ou síncope, que se submeteram a TAVI com: (1) válvula aórtica nativa ao ecocardiograma transtorácico (EcoTT), com a presença mínima de um dos critérios: gradiente transvalvar aórtico médio > 40 mmHg, ou velocidade do jato aórtico > 4 m/s, ou área valvar aórtica (AVA) < 1 cm^2^ (ou AVA indexada pela superfície corporal < 0,6 cm^2^/m^2^);^6^ (2) alto risco para cirurgia de troca da válvula aórtica (CTVA) definido pela equipe cardiológica; e (3) viabilidade de acesso vascular: transfemoral (TF), transubclávia (TSC) e transaórtico.

Este estudo foi conduzido de acordo com os princípios estabelecidos na Declaração de Helsinki e revistos em 2000 (Escócia 2000) e aprovado pelo Comitê de Ética em Pesquisa do Hospital Pró-Cardíaco sob o n° 423. Todos os pacientes assinaram o Termo de Consentimento Livre e Esclarecido.

### Procedimentos de investigação

Foram estudadas variáveis demográficas, da técnica do procedimento e do pós- procedimento correlacionadas a parâmetros clínicos e laboratoriais referentes à resposta inflamatória após TAVI.

Os exames laboratoriais incluíram hemograma completo, creatinina e PCR. Amostras de conveniência foram enviadas ao laboratório de análises clínicas e os resultados imediatamente disponibilizados. A dosagem de PCR ultrassensível sérica foi realizada por imunoensaio turbidimétrico na rotina laboratorial hospitalar (valor de referência adotado < 0,5 mg/dL) com o equipamento *Dimension EXL 200 Clinical Chemistry System* (Siemens, Alemanha).

Os procedimentos foram realizados sob sedação consciente com monitorização por EcoTT ou anestesia geral com monitorização por ecocardiograma transesofágico tridimensional (EcoTE). O acesso vascular foi realizado de forma cirúrgica. As próteses utilizadas foram: a autoexpansível Medtronic CoreValve bioprosthesis (Medtronic, Minneapolis, EUA) e a expansível por balão Edwards-Sapien XT (Edwards Lifesciences, Irvine, EUA).

A população estudada foi acompanhada por 1 ano após o implante. Os eventos adversos da fase pós-hospitalar foram coletados por meio de contatos telefônicos sistemáticos com os pacientes e/ou seus familiares e/ou seus médicos assistentes, e também por meio de laudos de exames e registros de hospitalizações e intervenções posteriores. Os contatos telefônicos e os registros do seguimento foram realizados aos 30 dias, 180 dias, e em 1 ano. Houve apenas um caso de perda de seguimento em 1 ano.

As definições do sucesso do implante do dispositivo e complicações seguiram a proposta do Valve Academic Research Consortium: foi considerado como sucesso o implante de apenas uma prótese, gradiente transvalvar médio final < 20 mm Hg, área efetiva do orifício valvar indexada > 0,85 cm^2^/m^2^ (> 0,7 cm^2^/m^2^ em pacientes com índice de massa corporal > 30 kg/m^2^), regurgitação aórtica <2+/4 e sobrevivência em 30 dias. A síndrome da resposta inflamatória sistêmica (SRIS) foi diagnosticada pela presença de dois ou mais dos critérios: febre (> 38°C), taquicardia (> 90 batimentos/minuto), taquipneia (> 20 respirações/minuto) e leucocitose (> 12000 leucócitos/mL).

Eventos CVs foram definidos como óbito CV ou de causa súbita e indeterminada; hospitalização por qualquer causa relacionada ao sistema cardiovascular, seja por arritmias, descompensações de insuficiência cardíaca, doença coronariana, intervenções percutâneas ou cirúrgicas; infarto agudo do miocárdio; realização de angioplastia coronariana; e acidente vascular encefálico isquêmico ou hemorrágico.

### Análise estatística

A análise descritiva foi apresentada sob a forma de tabelas e os dados observados expressos pela mediana e intervalo interquartílico (Q1 e Q3) para dados numéricos, e frequência (n) e percentual (%) para dados categóricos, além de alguns gráficos ilustrativos.

A análise inferencial foi composta pelos seguintes métodos: (1) na análise univariada para verificar a associação dos dados clínicos e cardiológicos com a sobrevida em 1 ano, foi utilizada a regressão de Cox individualmente; (2) na análise multivariada para identificar os preditores independentes para o desfecho óbito até 1 ano de acompanhamento, foi aplicada a regressão de Cox com o método de seleção das variáveis avançar passo a passo (*stepwise forward*)*;* (3) a curva de Kaplan-Meier foi construída para ilustrar a sobrevida em 1 ano estratificada por subgrupos da PCR pós e comparadas pela estatística de *log-rank;* (4) na análise univariada para verificar a associação dos dados clínicos e cardiológicos com a sobrevida em 1 ano entre os sobreviventes após alta hospitalar, foi utilizada a regressão de Cox individualmente; (5) na análise multivariada para identificar os preditores independentes para os desfecho óbito entre os sobreviventes após alta hospitalar até 1 ano de acompanhamento, foi aplicada a regressão de Cox com o método de seleção das variáveis avançar passo a passo (*stepwise forward*); e (6) foi realizada uma análise adicional, ao final, incluindo apenas os pacientes sobreviventes à fase intra-hospitalar, com análise multivariada para identificar os preditores independentes para o desfecho óbito até 1 ano de acompanhamento, utilizando-se a regressão de Cox com o método de seleção das variáveis avançar passo a passo (*stepwise forward*).

Foram aplicados métodos não paramétricos, pois todas as variáveis, em pelo menos um dos subgrupos, não apresentaram distribuição normal (Gaussiana), pela rejeição da hipótese de normalidade segundo o teste de Shapiro-Wilks. O nível de significância estatística adotado foi valor de p < 0,05. A análise estatística foi processada pelo software estatístico SAS System, versão 6.11 (SAS Institute, Inc., Cary, EUA).

## Resultados

### Características da população

Entre julho de 2009 e maio de 2015, 130 pacientes foram submetidos a TAVI em válvula nativa um único hospital privado e acompanhados por 1 ano.

As características demográficas e clínicas da população estudada estão descritas na Tabela 1. A creatinina sérica inicial foi 1,1 (0,9-1,4) mg/dL e o *clearance de creatinina estimado foi de 48,0 (21,8) mL/min pela fórmula* Cockcroft-Gault. A hemoglobina inicial foi 11,9 (10,4-13,1) mg/dL. Nove (6,9%) pacientes receberam hemotransfusão antes do procedimento.

**Tabela 1:** Características demográficas e clínicas da população estudada

Características	n = 130 n (%)
Idade (anos) (mediana)	83,0 (80,0-87,0)
Sexo masculino	67 (51,5)
IMC (mediana)	25,3 (22,5-29,4)
Quadro clínico	
Síncope	38 (29,2)
Angina do peito	27 (20,8)
IC classe funcional NYHA	
II	6 (4,8)
III	70 (53,8)
IV	54 (41,5)
Hipertensão arterial sistêmica	94 (72,3)
Diabetes mellitus	48 (36,9)
Doença arterial coronariana	70 (53,8)
IAM prévio	15 (11,5)
CRVM prévia	30 (23,1)
ICP prévia	42 (32,3)
AVE prévio	7 (5,4)
Doença vascular periférica	31 (23,8)
DPOC	12 (9,2)
Doença renal crônica*	101 (77,7)
Hipertensão arterial pulmonar	40 (30,8)
Marca-passo prévio	25 (19,2)
STS mortalidade (%)	8,6 (4,8-19,3)
STS morbidade (%)	34,6 (24,8-63,1)
Anemia	83 (63,8)
Fibrilação atrial	17 (13,1)
Disfunção do VE (FEVE<50%)	33 (25,4)

IMC: índice de massa corporal; IC: insuficiência cardíaca; IAM: infarto agudo do miocárdio; CRVM: cirurgia de revascularização miocárdica; ICP: intervenção coronariana percutânea; AVE: acidente vascular encefálico; NYHA: New York Heart Association; DPOC: doença pulmonar obstrutiva crônica; STS: Society of Thoracic Surgeons; VE: ventrículo esquerdo; FEVE: fração de ejeção do VE.

* Taxa de filtração glomerular estimada pela fórmula Cockcroft-Gault < 60 mL/min.

Ao EcoTT inicial, a AVA foi 0,6 (0,6-0,8) cm^2^ e o gradiente VE-Ao médio 45,5 (34,0-57,3) mmHg. A presença de insuficiência aórtica moderada ou grave associada ocorreu em 14 (10,8%) casos. A fração de ejeção do VE (método Simpson) foi de 64,0% (48,0-73,0%).

Valvuloplastia aórtica por balão e intervenção coronariana percutânea (ICP) dias antes do TAVI foi realizado em 4 (3,1%) e em 13 (10,0%) pacientes, respectivamente.

Os procedimentos foram realizados sob anestesia geral em 80,8% dos casos. O acesso vascular foi realizado por TF em 123 (94,6%) pacientes, TSC em 6 (4,6%), e transaórtico em 1 paciente (0,8%). A realização de ICP no mesmo tempo cirúrgico do TAVI ocorreu em 8 (6,2%) casos. A pré-dilatação da válvula foi realizada em 107 (82,3%) pacientes. A prótese CoreValve foi implantada em 132 (97,0%) pacientes, e a prótese Edwards-Sapien XT em 4 (3,0%). O número de “corridas” de *rapid* *pacing* foi 1,0 (1,0-2,0). Manobras para a correção de regurgitação paraprotética foram realizadas em 43 pacientes, sendo: pós-dilatação em 38 (36,9%) casos, implante de segunda válvula em 4 (3,1%), e tração por laço em 1 (0,8%) caso.

O gradiente VE-Ao médio ao EcoTT inicial foi reduzido de 45,5 (34,0-57,3) mmHg para 7,0 (5,0-10) mmHg (p < 0,001) após o procedimento. Ao final foi encontrada regurgitação paraprotética moderada em 7 (5,4%) pacientes.

A necessidade de implante de novo marca-passo definitivo ocorreu em 30 (23,1%) casos. Complicações vasculares maiores ocorreram em 7 (5,4%) pacientes. Hemotransfusões ocorreram em 28 (21,5%) pacientes, dos quais 10 (7,6%) receberam 1 unidade de hemácias (UH), 9 (6,9%) receberam de 2 a 3 UHs, e 9 (6,9%) receberam 4 UHs ou mais.

Insuficiência renal aguda (IRA) foi encontrada em 31 (23,8%) pacientes, ocorrendo em 25 (19,5%), 4 (3,1%) e 2 (1,6%) para os estágios I, II e III, respectivamente, nas primeiras 72 horas. Hemodiálise foi realizada em 5 (3,9%) pacientes ao longo da internação. As plaquetas variaram de 194 (158-237) mil/mm^3^ para 135 (101-165) mil/mm^3^ com nadir em 72 horas (p<0,0001).

O sucesso do implante do dispositivo ocorreu em 115 (88,5%) pacientes. O tempo de internação hospitalar após TAVI foi 7 (6-7) dias, variando de 3-212 dias.

A mortalidade intra-hospitalar ocorreu em 8 (6,2%) pacientes, com 1 caso de óbito após 30 dias por sépsis

### Resposta inflamatória antes e após TAVI

A SRIS foi identificada em 55 (42,6%) pacientes. Infecções urinárias ou respiratórias foram tratadas com uso de antibióticos em 13 (10,0%) pacientes. Hemoculturas ou urinoculturas foram positivas em 4 casos.

A leucometria variou de 6675 (5535-8623) células/mm^3^ no basal para 10520 (8570-13800) células/mm^3^ no pico em 24 horas após TAVI (p < 0,001).

**Figura 1:**
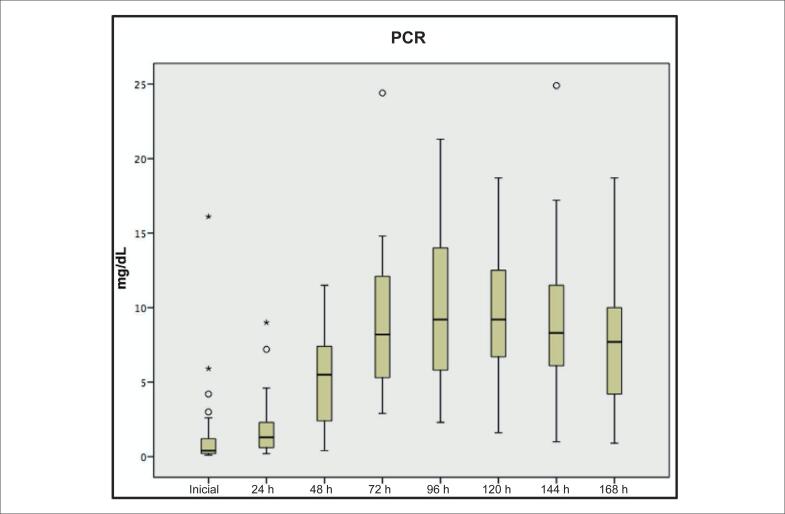
Dosagem da PCR na primeira semana. PCR: proteína C-reativa.

O PCR inicial foi 0,3 (0,2-1,0) mg/dL, sendo que 41 (34,5%) pacientes apresentaram PCR elevada (> 0,5 mg/dL). O pico de PCR foi 7,0 (5,3-12,1) mg/dL e ocorreu no quarto dia após TAVI (Figura 1).

### Seguimento de 30 dias e 1 ano

No seguimento de 30 dias, a mortalidade ocorreu em 7 (5,4%) pacientes. Houve reinternação em 10 (7,8%) casos, dos quais 8 por eventos CV.

A mortalidade global em 1 ano foi de 14,6%. As causas de óbito foram CV em 8 (42,0%) pacientes e não CV em 11 (58,0%), sendo que neste último houve predomínio de sepsis (n = 9).

**Figura 2:**
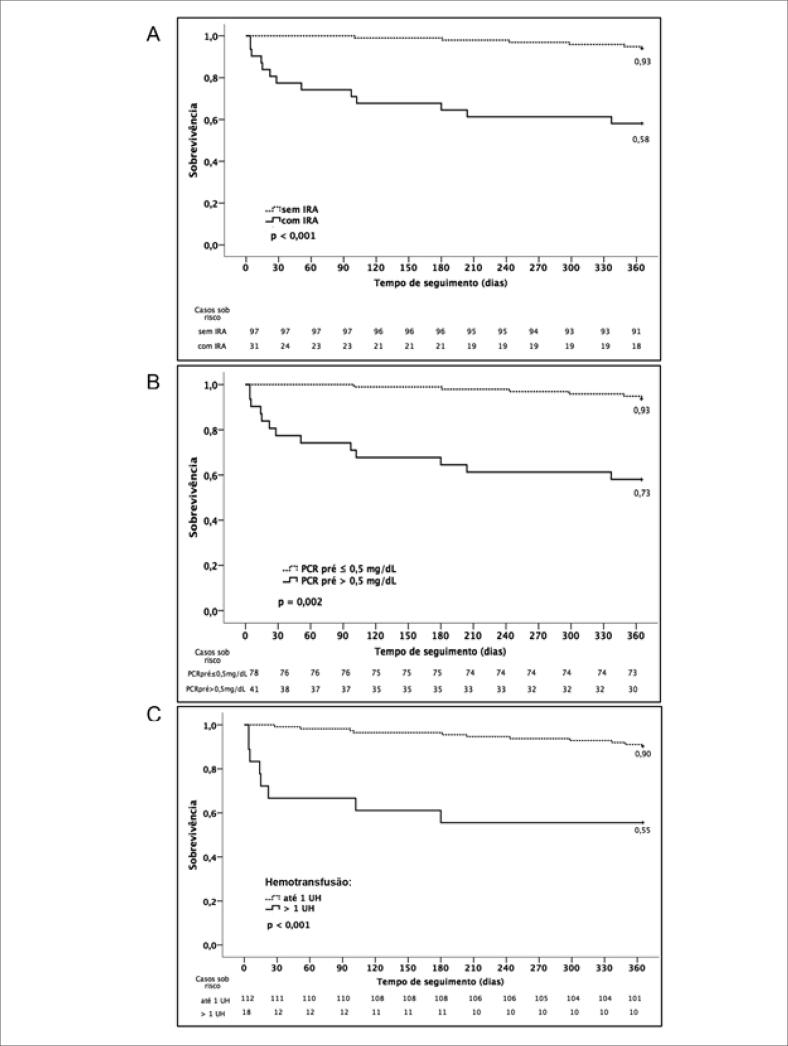
Sobrevivência em 1 ano estratificada por (A) presença de IRA, (B) PCR inicial > 0,5 mg/dL e (C) hemotransfusão > 1 UH. PCR: proteína C-reativa; IRA: insuficiência renal aguda; UH: unidade de hemácias.

Foi realizada uma comparação entre os casos de sobreviventes e de óbitos em 1 ano (Tabela 2). Os preditores independentes de mortalidade em 1 ano foram presença de IRA, PCR inicial elevado e hemotransfusão ≥ 4 UH (Tabela 3), e as curvas de sobrevivência em 1 ano estratificadas para estas variáveis foram demonstradas na Figura 2. Quando avaliamos apenas os pacientes após alta hospitalar, observamos que PCR inicial > 0,5 mg/dL permaneceu como preditora independente de óbito em 1 ano (Tabela 4).

**Tabela 2:** Características dos grupos óbito e sobreviventes em 1 ano

Características		Óbito 1 ano n = 19	Vivos 1 ano n = 111	RR (IC 95%)	Valor de p
Idade (anos)		84 (81-87)	83 (80-87)	-	0,3
Sexo masculino		36,8%	54,1%	-	0,2
IMC (kg/m2)		26,2 (22,6-27,4)	25,2 (22,5-30,1)	-	0,8
IC CF NYHA IV		57,9%	38,7%	-	0,1
Diabetes mellitus		42,1%	36,0%	-	0,6
DAC		42,1%	36%	-	0,6
DVP		26,3%	23,4%	-	0,4
DPOC		15,8%	8,1%	-	0,3
Escore STS (%)		17,9 (8,1-30,2)	8,1 (4,7-17,1)	1,03 (1,01-1,06)	0,02
Creatinina inicial (mg/dL)		1,3 (0,8-1,5)	1,1 (0,9-1,3)	-	0,8
FEVE (%)		55 (31-73)	64 (50,5-73,0)	0,98 (0,95-1,00)	0,04
Hemoglobina inicial (mg/dL)		11,2 (10,2-12,9)	12,0 (10,6-13,3)	-	0,4
Hemoglobina nadir (mg/dL)		8,1 (7,4-9,9)	9,8 (8,4-10,9)	0,68 (0,49-0,94)	0,01
PCR inicial (mg/dL)		1,5 (0,2-2,8)	0,3 (0,2-0,9)	1,19 (1,06-1,34)	<0,0001
PCR inicial > 0,5 mg/dL		68,8%	29,1%	4,70 (1,63-13,5)	0,004
PCR pico pós-TAVI (mg/dL)		14,3 (6,0-16,2)	7,8 (5,1-11,1)	1,14 (1,06-1,22)	<0,0001
SRIS		47,3%	41,8%	-	0,6
IAo pos TAVI ≥ +2/4		5,3%	5,4%	-	0,99
Complicação vascular maior		10,5%	4,5%	-	0,2
Sangramento	Maior	26,3%	20,7%	-	0,2
	Risco à vida	26,3%	4,5%	7,85 (2,62-23,5)	<0,001
Hemotransfusão	2 a 3 UH	15,8%	5,5%	4,3 (1,20-15,5)	0,02
	≥ 4 UH	26,3%	3,6%	9,4 (3,24-27,2)	<0,001
	Estágio I	52,6%	58,2%	8,2 (3,0-23)	<0,001
IRA	Estágio II	10,5%	1,8%	14,4 (2,9-72)	0,001
	Estágio III	5,3	0,9	14,7 (1,8-123)	0,013
Novo marca-passo		42,1%	19,8%	2,72 (1.09-6,8)	0,03
IC CF NYHA III 30 dias		38,5%	0,0%	66,8 (16-279)	<0,001

RR: razão de risco; IC: intervalo de confiança; IMC: índice de massa corporal; IC: insuficiência cardíaca; NYHA: New York Heart Association; DAC: doença arterial coronariana; DVP: doença vascular periférica DPOC: doença pulmonar obstrutiva crônica; FEVE: fração de ejeção do ventrículo esquerdo; STS: Society of Thoracic Surgeons; IRA: insuficiência renal aguda; PCR: proteína C-reativa; IAo: insuficiência aórtica; SRIS: síndrome da resposta inflamatória sistêmica; CF: classe funcional; TAVI: implante de válvula aórtica transcateter (transcatheter aortic valve implantation); UH: unidade de hemácias. Foram aplicados os testes de Mann-Whitney (variáveis numéricas) e qui-quadrado ou exato de Fisher (variáveis categóricas).

**Tabela 3:** Análise multivariada segundo a regressão de Cox para óbito em 1 ano

Variáveis no modelo	Coeficiente	EP	RR	IC 95%	Valor de p
IRA	1,983	0,624	7,43	2,1-24,7	0,001
PCR inicial > 0,5 mg/dL	1,422	0,577	4,15	1,3-12,9	0,01
Hemotransfusão ≥ 4 UH	1,543	0,649	4,68	1,3-16,7	0,02

EP: erro-padrão do coeficiente; RR: razão de risco; IC: intervalo de confiança; IRA: insuficiência renal aguda; PCR: proteína C-reativa; UH: unidade de hemácias. Método de seleção de variáveis utilizado: stepwise forward

**Tabela 4:** Análise multivariada segundo a regressão de Cox para óbito em 1 ano nos pacientes com alta hospitalar

Variáveis no modelo	coeficiente	EP	RR	IC 95%	Valor de p
IC CF NYHA III 30 dias	3,3	1,0	27,5	3,8-199	0,001
Sexo masculino	-4,1	1,3	0,02	0,001-0,23	0,002
Novo marca-passo	2,3	0,8	10,2	2,0-52,3	0,005
PCR inicial >0,5 mg/dL	2,1	0,8	8,9	1,6-48,0	0,01

EP: erro-padrão do coeficiente; RR: razão de risco; IC: intervalo de confiança. IRA: insuficiência renal aguda; PCR: proteína C-reativa; UH: unidade de hemácias. Método de seleção de variáveis utilizado: stepwise forward.

A comparação entre os grupos com PCR inicial elevada (> 0,5 mg/dL) e normal é demonstrada na Tabela 5.

**Tabela 5:** Características dos grupos com PCR inicial > 0,5 mg/dL e ≤ 0,5 g/dL

Características		PCR inicial > 0,5 mg/dL n = 46	PCR inicial ≤ 0,5 mg/dL n = 84	Valor de p
Idade (anos)		84 (80-88)	83 (80-87)	0,3
Sexo masculino		53,7%	48,7%	0,6
IMC (kg/m^2^)		25,5 (23,3-27,2)	25,3 (22,2-30,1)	0,7
IC CF NYHA IV		61,0%	29,5%	0,001
Diabetes mellitus		31,7%	38,5%	0,5
DAC		58,5%	51,3%	0,4
DVP		26,8%	23,1%	0,6
DPOC		14,6%	5,1%	0,09
Escore STS (%)		18,8 (7,7-26,6)	6,9 (4,2-15,3)	0,001
FEVE (%)		60 (44-68)	66 (52-74)	0,3
Creatinina inicial (mg/dL)		1,3 (0,9-1,5)	1,1 (0,9-1,3)	0,06
Hemoglobina inicial (mg/dL)		11,8 (10,0-13,2)	12,1 (10,9-13,3)	0,2
Hemoglobina nadir (mg/dL)		9,3 (8,0-10,9)	9,9 (8,4-10,6)	0,5
Plaquetas inicial (x10^3^/mm^3^)		200 (145- 287)	194 (165-226)	0,4
Plaquetas nadir (x10^3^/mm^3^)		149 (101-192)	125 (102-152)	0,04
Pico de PCR (mg/dL)		11,5 (6,5-14,8)	7,2 (4,6-10,3)	0,002
IAo pós TAVI ≥ +2/4		2,4%	6,4%	0,6
Sangramento	Maior	14,6%	25,6%	0,2
	Risco à vida	9,8%	6,4%	
Hemotransfusão	2 a 3 UH	9,8%	3,8%	0,4
	≥ 4 UH	7,3%	6,4%	
	Sem IRA	66,7%	80,8%	0,04
IRA	Estágio I	25,6%	17,9%	
	Estágio II	7,7%	0%	
	Estágio III	0%	1,3%	
SRIS		47,5%	42,3%	0,6
Novo marcapasso		26,8%	21,8%	0,6

IMC: índice de massa corporal; IC: insuficiência cardíaca; NYHA: New York Heart Association; CF: classe funcional; DVP: doença vascular periférica; DPOC: doença pulmonar obstrutiva crônica; STS: Society of Thoracic Surgeons; FEVE: fração de ejeção do ventrículo esquerdo; PCR: proteína C-reativa; TAVI: implante de válvula aórtica transcateter; IAo: insuficiência aórtica; IRA: insuficiência renal aguda; SRIS: síndrome da resposta inflamatória sistêmica; DAC: doença arterial coronariana; UH: unidade de hemácias. Foram aplicados os testes de Mann-Whitney (variáveis numéricas) e qui-quadrado ou exato de Fisher (variáveis categóricas).

O valor de PCR ≥ 10,0 mg/dL no pico exibiu sensibilidade de 64,7% e especificidade de 69,2% para mortalidade ao final de 1 ano na curva ROC, com area sob a curva = 0,71 [intervalo de confiança (IC) de 95%, 0,57-0,86; p = 0,005]. A PCR no pico após TAVI foi preditora de mortalidade em 1 ano apenas na análise univariada, com razão de risco (RR) =1,14 (IC95%,1,06-1,22; p < 0,0001).

## Discussão

Este estudo avaliou o impacto da resposta inflamatória na mortalidade do primeiro ano pós-TAVI através da dosagem de PCR no peroperatório, com predomínio de implantação da válvula CoreValve pela via TF. A inflamação crônica de baixa intensidade (PCR > 0,5 mg/dL) antes do TAVI ocorreu em um terço dos pacientes, sendo fator preditor independente de mortalidade no primeiro ano (RR = 4,1; p = 0,01). O pico de elevação da PCR ocorreu entre o terceiro e o quarto dias, sendo que valores de pico de PCR ≥ 10 mg/dL associaram-se à maior mortalidade, mas foram influenciados pela ocorrência de IRA e por hemotransfusões volumosas.

A avaliação do prognóstico através de biomarcadores de inflamação pré-TAVI também foi realizado por Sinning et al.,^7^ que relataram que o biomarcador de inflamação GDF-15 e o escore de risco cirúrgico EuroSCORE II foram os melhores preditores de mortalidade no primeiro ano pós-TAVI. Naquele trabalho, a PCR pré-TAVI acarretou maior risco para óbito (RR = 1,2; IC95%, 1,0-1,4; p = 0,012). De forma semelhante, encontramos que a PCR mediana pré- TAVI indicou maior risco de morte ao final de 1 ano (RR = 1,2; IC95%, 1,0-1,3; p < 0,001). Entretanto, julgamos que a análise da PCR como variável categórica mostrou-se mais útil, principalmente ao adotar o valor de corte (> 0,5 mg/dL) baseado em publicações que envolveram cirurgia cardíaca^8^ e, mais recentemente, TAVI.^9,10^

A PCR elevada no pré-operatório de cirurgias cardíacas foi associada a maior mortalidade no estudo de Cappabianca et al.,^8^ que avaliaram a PCR no pré-operatório de 597 pacientes submetidos a diferentes tipos de cirurgia cardíaca (CTVA em 15%) e observaram que aqueles com PCR >0,5 mg/dL evoluíram com maior mortalidade em 3 anos de seguimento (*odds* *ratio* [OR], 1,93; p=0,05). O valor normal da PCR < 0,3 mg/dL foi proposto a partir de um estudo epidemiológico que avaliou eventos CV sem a realização de procedimentos invasivos e possivelmente não represente o melhor valor de corte no contexto de cirurgias.

Neste estudo, a PCR inicial elevada foi associada à IC descompensada, observando-se maior proporção de pacientes em classe funcional IV e níveis mais elevados de *brain natriuretic peptide* (BNP). Villacorta et al.,^11^ descreveram que pacientes com disfunção sistólica do VE e IC descompensada apresentaram PCR mais elevada na internação. Jensen et al. descreveram a relação da PCR com o BNP na IC descompensada.^12^ Entretanto é pouco plausível que o pior prognóstico relacionado à PCR possa ser atribuído exclusivamente à sua relação com a IC uma vez que mais da metade das mortes foi de causa não CV.

A PCR cronicamente elevada também tem sido descrita entre idosos, com crescentes evidências de que a inflamação sistêmica crônica tem impacto na qualidade de vida e na sobrevida. O termo *inflammaging* foi proposto para descrever inúmeras condições relacionadas à presença de inflamação nos idosos.^13^ Numa metanálise, foram identificados 20 biomarcadores circulantes no sangue com potencial para avaliação prognóstica em idosos, sendo a PCR foi preditora de mortalidade global (RR = 1,4; p<0,001) e mortalidade CV (RR = 1,3; p=0,03).^14^ No presente estudo não foi observada relação entre PCR e idade mais avançada, mas observou-se que no grupo com PCR inicial elevada o escore STS foi maior (19% vs 7%; p = 0,001), o que sugere se correlacionar com a saúde global dos pacientes. O grupo com PCR pré-TAVI > 0,5 mg/dL teve evolução intra-hospitalar com maior pico na elevação da PCR e IRA, além de plaquetopenia mais intensa.

Após o TAVI, a cinética da PCR em resposta ao procedimento na primeira semana mostrou pico de elevação entre 72-96 horas, com valores ainda elevados até o sétimo dia, semelhante a outros estudos.^9,15^ A cinética da PCR nos pacientes submetidos a TAVI por via TF é diferente daquela encontrada na CTVA.

Tradicionalmente, o valor de pico de um biomarcador de inflamação é considerado a resposta inflamatória máxima obtida. No curto prazo, o valor de pico da PCR foi avaliado por Krumsdorf et al.,^9^ que observaram em análise univariada que a PCR ≥ 10 mg/dL foi associada à maior mortalidade em 30 dias. Esse achado no curto prazo não foi confirmado por Ruparelia et al.,^15^ No longo prazo, o valor prognóstico da elevação da PCR não foi ainda descrito. No presente trabalho encontrou-se que o pico de PCR ≥ 10mg/dL foi capaz de predizer óbito no primeiro ano (RR = 3,74; p = 0,009), entretanto esta variável não foi fator independente.

Na presente casuística, não foi encontrada associação entre alguns aspectos técnicos e o grau de inflamação, como: o número de corridas de *rapid pacing* ou o implante direto (sem pré-dilatação por balão antes do TAVI). Sinning et al.,^16^ observaram correlação entre SRIS e o número de corridas de *rapid pacing* e/ou pós-dilatação. Ruparelia et al.^15^ encontraram PCR pico no terceiro dia mais elevado entre aqueles com pré-dilatação [11,0 (0,8) mg/dL vs 5,1 (0,3) mg/dL; p < 0,001).

Neste estudo, os fatores preditores independentes pós-operatórios de mau prognóstico em 1 ano foram a IRA e a hemotransfusão volumosa (≥ 4 UH), confirmado por outros autores.^17,18^ A mortalidade de causas CV (42%) foi quase tão frequente quanto causas de óbito não CV (58%), o que também foi decrito do estudo PARTNER.^19^

A avaliação de fatores prognósticos relativos ao TAVI tem inúmeras implicações que estão relacionadas desde a estratégia cirúrgica até a avaliação da futilidade do procedimento. A contribuição deste trabalho pode auxiliar outros estudos na comparação de técnicas e próteses valvares. É importante ressaltar que as próteses utilizadas neste estudo já foram substituídas por novas versões, que necessitam bainhas introdutoras menores, em um curto período de tempo, o que deverá reduzir complicações vasculares. Portanto, este estudo poderá servir como parâmetro para futuras comparações.

O presente estudo tem limitações relacionadas à natureza observacional, retrospectiva e não consecutiva de seu desenho. Embora a casuística represente umas das maiores experiências nacionais unicêntricas, o tamanho da amostra é pequeno, se comparado a estudos internacionais multicêntricos, e a avaliação de eventos CVs não contou com centro de adjudicação de eventos. A dosagem do PCR e do BNP no seguimento após alta poderia esclarecer a relação entre IC e inflamação valvar, assim como o potencial papel inflamatório da permanência dos folhetos valvares não ressecados que ficaram encarcerados pela prótese valvar implantada.

## Conclusões

A PCR >0,5 mg/dL pré-TAVI está presente em um terço dos casos e mostrou-se fator preditor independente de mortalidade no primeiro ano, assim como a ocorrência de IRA e hemotransfusões volumosas. O pico de PCR ocorre entre o terceiro e o quarto dias pos-TAVI e, quando atinge ≥ 10 mg/dL, se correlacionou com maior mortalidade em 1 ano, embora seja dependente de outros fatores como IRA e hemotransfusão.

## References

[1] Brasil. Presidência da República. Secretaria de Direitos Humanos. Secretaria Nacional de Promoção Defesa dos Direitos Humanos. [Internet]. Dados sobre o envelhecimento no Brasil. [acesso em 2016 set. 21]. Disponível em:<http://www.sdh.gov.br/assuntos/pessoa-idosa/dados-estatisticos/DadossobreoenvelhecimentonoBrasil.pdf>

[2] Franceschi C, Campisi J. Chronic inflammation (inflammaging) and its potential contribution to age-associated diseases. J Gerontol A Biol Sci Med Sci. 2014;69(Suppl 1):S4-9. 10.1093/gerona/glu057 24833586

[3] Wu IC, Lin CC, Hsiung CA. Emerging roles of frailty and inflammaging in risk assessment of age-related chronic diseases in older adults: the intersection between aging biology and personalized medicine. Biomedicine (Taipei). 2015;5(1):1. 10.7603/s40681-015-0001-1 PMC4333299 25722960

[4] Otto CM, Kuusisto J, Reichenbach D, Gown A, O’Brien KD. Characterization of the early lesion of ‘degenerative’ valvular aortic stenosis. Histological and immunohistochemical studies. Circulation. 1994;90(2):844-53. 10.1161/01.cir.90.2.844 7519131

[5] Mazzone A, Epistolato MC, De Caterina R, Storti S, Vittorini S, Sbrana S, et al. Neoangiogenesis, T-lymphocyte infiltration, and heat shock protein-60 are biological hallmarks of an immunomediated inflammatory process in end-stagecalcified aortic valve stenosis. J Am Coll Cardiol. 2004;43(9):1670-6. 10.1016/j.jacc.2003.12.041 15120829

[6] Kappetein AP, Head SJ, Généreux P, Piazza N, van Mieghem NM, Blackstone EH, et al. Updated standardized endpoint definitions for transcatheter aortic valve implantation: the Valve Academic Research Consortium-2 consensus document. J Am Coll Cardiol. 2012;60(15):1438-54. 10.1016/j.jacc.2012.09.001 23036636

[7] Sinning JM, Wollert KC, Sedaghat A, Widera C, Radermacher MC, Descoups C, et al. Risk scores and biomarkers forthe prediction of 1-year outcome after transcatheter aortic valve replacement. Am Heart J. 2015;170(4):821-9. 10.1016/j.ahj.2015.07.003 26386807

[8] Cappabianca G, Paparella D, Visicchio G, Capone G, Lionetti G, Numis F, et al. Preoperative C-reactive proteinpredicts mid-term outcome after cardiac surgery. Ann Thorac Surg. 2006;82(6):2170-8. 10.1016/j.athoracsur.2006.06.039 17126130

[9] Krumsdorf U, Chorianopoulos E, Pleger ST, Kallenbach K, Karck M, Katus HA, et al. C-reactive protein kinetics and its prognostic value after transfemoral aortic valve implantation. J Invasive Cardiol. 2012;24(6):282-6. 22684383

[10] Stähli BE, Grünenfelder J, Jacobs S, Falk V, Landmesser U, Wischnewsky MB, et al. Assessment of inflammatory response to transfemoral transcatheter aortic valve implantation compared to transapical and surgical procedures: a pilot study. J Invasive Cardiol. 2012;24(8):407-11. 22865312

[11] Villacorta H, Masetto AC, Mesquita ET. C-reactive protein: an inflammatory marker with prognostic value in patients with decompensated heart failure. Arq Bras Cardiol. 2007;88(5):585-9. 10.1590/s0066-782x2007000500014 17589635

[12] Jensen J, Ma LP, Fu ML, Svaninger D, Lundberg PA, Hammarsten O. Inflammation increases NT-proBNP and the NT-proBNP/BNP ratio. Clin Res Cardiol. 2010;99(7):445-52. 10.1007/s00392-010-0140-z 20229122

[13] Franceschi C, Capri M, Monti D, Giunta S, Olivieri F, Sevini F, et al. Inflammaging and anti-inflammaging: a systemic perspective on aging and longevity emerged from studies in humans. Mech Ageing Dev. 2007;128(1):92-105. 10.1016/j.mad.2006.11.016 17116321

[14] Barron E, Lara J, White M, Mathers JC. Blood-borne biomarkers of mortality risk: systematic review of cohort studies. PloS One. 2015;10(6):e0127550. 10.1371/journal.pone.0127550 PMC4454670 26039142

[15] Ruparelia N, Panoulas VF, Frame A, Ariff B, Sutaria N, Fertleman M, et al. Impact of clinical and procedural factors upon C-reactive protein dynamics following transcatheter aortic valve implantation. World J Cardiol. 2016;8(7):425-31. 10.4330/wjc.v8.i7.425 PMC4958693 27468335

[16] Sinning JM, Scheer AC, Adenauer V, Ghanem A, Hammerstingl C, Schueler R, et al. Systemic inflammatory response syndrome predicts increased mortality in patients after transcatheter aortic valve implantation. Eur Heart J. 2012;33(12):1459-68. 10.1093/eurheartj/ehs002 22285582

[17] Tchetche D, Van der Boon RM, Dumonteil N, Chieffo A, Van Mieghem NM, Farah B, et al. Adverse impact of bleeding and transfusion on the outcome post- transcatheter aortic valve implantation: insights from the Pooled-RotterdAm-Milano-Toulouse In Collaboration Plus (PRAGMATIC Plus) initiative. Am Heart J. 2012;164(3):402-9. 10.1016/j.ahj.2012.07.003 22980308

[18] Seiffert M, Conradi L, Terstesse AC, Koschyk D, Schirmer J, Schnabel RB, et al. Blood transfusion is associated with impaired outcome after transcatheter aortic valve implantation. Catheter Cardiovasc Interv. 2015;85(3):460-7. 10.1002/ccd.25691 25292388

[19] Svensson LG, Blackstone EH, Rajeswaran J, Brozzi N, Leon MB, Smith CR, et al; PARTNER Trial Investigators. Comprehensive analysis of mortality among patients undergoing TAVR: results of the PARTNER trial. J Am Coll Cardiol. 2014;64(2):158-68. 10.1016/j.jacc.2013.08.1666 25011720

