# Supplementary material for: Proteína C-reativa como Marcador Prognóstico de Mortalidade no Primeiro Ano após Implante de Válvula Aórtica Transcateter em Estenose Aórtica
Source: Arq Bras Cardiol. 2021 Nov 1;117(5):1018–27. [Article in Portuguese] doi: 10.36660/abc.20190715 (PMC8682084; doi:10.36660/abc.20190715)
Supplement: Translation

# Translation: C-reactive Protein as a Prognostic Marker of 1-Year Mortality after Transcatheter Aortic Valve Implantation in Aortic Stenosis

**Authors:** André Luiz Silveira Sousa, Luiz Antônio Ferreira Carvalho, Constantino González Salgado, Rafael Lauria de Oliveira, Luciana Cristina Correia Lima e Lima, Nelson Durval Ferreira Gomes de Mattos, Francisco Eduardo Sampaio Fagundes, Alexandre Siciliano Colafranceschi, Evandro Tinoco Mesquita

**Affiliations:** 1 Niterói RJ Brazil Universidade Federal Fluminense - Hospital Antonio Pedro – Cardiologia, Niterói, RJ – Brazil; 2 Rio de Janeiro RJ Brazil Hospital Pró-Cardíaco – Hemodinâmica, Rio de Janeiro, RJ – Brazil

**Keywords:** C-Reactive Protein, Inflammation, Bomarkers, Heart Valve Prosthesis Implantation, Transcatheter Aortic Valve Replacement, Aortic Valve Stenosis

## Abstract

**Background:**

C-reactive protein (CRP) is an inflammation biomarker that can be a predictor of adverse events in cardiovascular procedures. Its use in the assessment of long-term prognosis of transcatheter aortic valve implantation (TAVI) is still incipient.

**Objective:**

To evaluate CRP as a prognostic marker in the first year after TAVI in aortic stenosis (AoS).

**Methods:**

CRP was assessed on the first postoperative week in a retrospective cohort of patients with AoS. Pre- and post- CRP levels were correlated with mortality, and predictors of 1-year mortality were investigated. Multivariate Cox regression was performed to identify independent factors of 1-year mortality.

**Results:**

This study evaluated 130 patients who underwent TAVI, with median age of 83 years, and 49% of women. High pre-TAVI CRP (> 0.5 mg/dL) was observed in 34.5% of the cases. Peak CRP was 7.0 (5.3-12.1) mg/dL no quarto dia. The rate of 1-year mortality was 14.5% (n = 19), being greater in the groups with high pre-TAVI CRP (68.8% vs 29.1%; p = 0,004) and with peak CRP ≥ 10.0 mg/dL (64.7% vs 30.8%; p = 0,009). Independent predictors of mortality were acute renal failure (ARF) (hazard ratio [HR] = 7.43; 95% confidence interval [95%CI], 2.1-24.7; p = 0,001), high pre-TAVI CRP (HR 4.15; 95%CI, 1.3-12.9; p = 0.01), and large blood transfusion [HR 4,68; 1,3-16,7; p = 0.02].

**Conclusions:**

High pre-TAVI CRP showed to be an independent predictor of 1-year mortality, as well as the presence of ARF and large blood transfusions.

## Introduction

Fibrocalcic aortic stenosis (AoS) is a degenerative disease whose number of cases is estimated to triplicate in Brazil in the next 20 years, due to population aging.^1^

Transcatheter aortic valve implantation (TAVI) is a treatment that has been increasingly used in older adults, a group affected by chronic low-grade systemic inflammation (inflammaging),^2^ whose presence is associated with greater: (1) organ dysfunction and frailty; (2) immune system compromise and risk of infections; and (3) rate of cardiovascular (CV) events and mortality.^3^ This systemic inflammation is compounded by aortic valve inflammation in the process of valve degeneration from the initial stage of lipid infiltration^4^ to the end-stage of calcification and neovascularization of leaflets.^5^ Therefore, both systemic and valve inflammations are present before TAVI and have an increase, at different levels, after the procedure, depending on the adopted techniques and strategies.

However, few studies have used biomarkers to assess the role of systemic inflammation in mid- and long-term prognosis after TAVI. The present study evaluated the extent of systemic inflammation before and over 1 week after TAVI through serum C-reactive protein (CRP) levels and correlated them with 1-year prognosis.

## Methods

### Population

This is a retrospective, cohort, observational study of symptomatic patients with severe AoS who underwent TAVI at a private hospital from June 2009 to May 2015. During this period, 137 TAVIs were performed on native valves, of which the following were excluded from the present study: four cases of mechanical complications during the procedure resulting in death within 24 hours, and three procedures performed in critically-ill patients. We investigated 130 patients with severe AoS and symptoms of heart failure (HF), angina, or syncope who underwent TAVI with: (1) native aortic valve on transthoracic echocardiogram (TTE), with the presence of at least one of the following criteria: mean aortic transvalvular gradient > 40 mm Hg, or aortic jet velocity > 4 m/s, or aortic valve area (AVA) < 1 cm^2^ (or AVA indexed by body surface < 0.6 cm^2^/m^2^);^6^ (2) high risk for surgical aortic valve replacement (SAVR) as defined by the cardiologic team; (3) viable vascular access: transfemoral (TF), trans subclavian (TSC), and transaortic.

This study was conducted in compliance with the principles set forth in the Declaration of Helsinki and reviewed in 2000 (Scotland 2000) and was approved by the Research Ethics Committee of Hospital Pró-Cardíaco under no. 423. All patients signed an Informed Consent Form.

### Investigation procedures

This study evaluated demographic variables and intervention and post-intervention variables correlated with clinical and laboratory parameters involved in inflammatory response after TAVI.

Laboratory tests included complete blood count, creatinine, and CRP. Convenience samples were sent to a clinical analysis laboratory, and results were immediately made available. Serum high-sensitive CRP concentrations were measured by turbidimetric immunoassay according to the hospital laboratory routine (reference value < 0.5 mg/dL) with the Dimension EXL 200 Clinical Chemistry System device (Siemens, German).

The procedures were performed under conscious sedation with TTE monitoring, or under general anesthesia with monitoring by three-dimensional transesophageal echocardiogram (TEE). Vascular access was obtained surgically. The following prostheses were used: self-expandable Medtronic CoreValve bioprosthesis (Medtronic, Minneapolis, USA) and balloon-expandable Edwards-Sapien XT valve (Edwards Lifesciences, Irvine, USA).

The study population was followed for 1 year after TAVI. Post-hospitalization adverse events were collected by systematic phone calls with the patients and/or their relatives and/or their treating physicians, as well as by test reports and records of subsequent hospitalizations and interventions. Phone calls and follow-up records were performed at 30 days, 180 days, and 1 year. However, only one patient was lost to follow-up at 1 year.

TAVI success and complications were defined according to the criteria proposed by the Valve Academic Research Consortium: TAVI success was considered the implantation of only one prosthesis, final mean aortic transvalvular gradient < 20 mm Hg, Indexed effective orifice area > 0.85 cm^2^/m^2^ (> 0.7 cm^2^/m^2^ in patients with body mass index > 30 kg/m^2^), aortic regurgitation < 2+/4, and survival at 30 days. Systemic inflammatory response syndrome (SIRS) was diagnosed by the presence of at least two of the following criteria: fever (> 38^o^C), tachycardia (> 90 beats/minute), tachypnea (> 20 breaths/minute), and leukocytosis (> 12000 leukocytes/mL).

CV events were defined as CV death or sudden undetermined death; hospitalization for any cause related to the CV system, such as arrhythmia, decompensated HF, coronary artery disease, percutaneous or surgical intervention; acute myocardial infarction; execution of coronary angioplasty; and ischemic or hemorrhagic stroke.

### Statistical analysis

Descriptive analysis was presented in tables, and the observed data were expressed as median and interquartile range (Q1 and Q3) for numeric data, and frequency (n) and percentage (%) for categorical data, in addition to some illustrative graphs.

Inferential analysis consisted of the following methods: (1) the association of clinical and cardiologic data with 1-year survival was assessed in an univariate analysis using an individual Cox regression model; (2) the independent predictors of 1-year mortality were identified in a multivariate analysis using Cox regression with stepwise forward selection of variables; (3) the Kaplan-Meier curves were built to illustrate 1-year survival stratified by post-TAVI CPR subgroups and compared by log-rank statistics; (4) the association of clinical and cardiologic data with 1-year survival among survivors after hospital discharge was assessed in an univariate analysis using an individual Cox regression model; (5) the independent predictors of 1-year mortality among survivors after hospital discharge were identified in a multivariate analysis using Cox regression with stepwise forward selection of variables; (6) finally, an additional analysis was conducted, including only the patients who survived hospitalization, with a multivariate analysis to identify the independent predictors of 1-year mortality, using Cox regression with stepwise forward selection of variables.

Non-parametric methods were used, because all variables did not have a normal (Gaussian) distribution in at least one of the subgroups, leading to the rejection of the normality hypothesis according to the Shapiro-Wilks test. The level of significance was set at p < 0.05. Statistical analysis was conducted by the SAS System statistical software, version 6.11 (SAS Institute, Inc., Cary, USA).

## Results

### Population characteristics

From July 2009 to May 2015, 130 patients underwent TAVI on native valve at a single private hospital and were followed for 1 year.

Demographic and clinical characteristics of the study population are described in Table 1. Baseline serum creatinine was 1.1 (0.9-1.4) mg/dL, and creatinine clearance was estimated at 48.0 (21.8) mL/min by the Cockcroft-Gault formula. Baseline hemoglobin was 11.9 (10.4-13.1) mg/dL. Nine (6.9%) patients received blood transfusion before the procedure.

**Table 1:** Demographic and clinical characteristics of the study population

Characteristics	N = 130 n (%)
Age (years) (median)	83.0 (80.0-87.0)
Male sex	67 (51.5)
BMI (median)	25.3 (22.5-29.4)
Clinical presentation	
Syncope	38 (29.2)
Angina pectoris	27 (20.8)
HF, NYHA functional class	
II	6 (4.8)
III	70 (53.8)
IV	54 (41.5)
Systemic arterial hypertension	94 (72.3)
Diabetes mellitus	48 (36.9)
Coronary arterial disease	70 (53.8)
Previous AMI	15 (11.5)
Previous MRS	30 (23.1)
Previous PCI	42 (32.3)
Previous stroke	7 (5.4)
Peripheral vascular disease	31 (23.8)
COPD	12 (9.2)
Chronic kidney disease*	101 (77.7)
Pulmonary arterial hypertension	40 (30.8)
Previous pacemaker	25 (19.2)
STS mortality (%)	8.6 (4.8-19.3)
STS morbidity (%)	34.6 (24.8-63.1)
Anemia	83 (63.8)
Atrial fibrillation	17 (13.1)
LV dysfunction (LVEF < 50%)	33 (25.4)

BMI: body mass index; HF: heart failure; NYHA: New York Heart Association; AMI: acute myocardial infarction; MRS: myocardial revascularization surgery; PCI: percutaneous coronary intervention; COPD: chronic obstructive pulmonary disease; STS: Surgeons Thoracic Society; LV: left ventricle LVEF: LV ejection fraction.

* Glomerular filtration rate estimated by the Cockcroft-Gault formula < 60 mL/min.

On baseline TTE, AVA was 0.6 (0.6-0.8) cm^2^, and mean left ventricle (LV)-aortic gradient was 45.5 (34.0-57.3) mm Hg. Associated moderate or severe aortic failure was present in 14 (10.8%) cases. LV ejection fraction (Simpson method) was 64.0% (48.0-73.0%).

Balloon aortic valvuloplasty and percutaneous coronary intervention (PCI) were performed days before TAVI in four (3.1%) and 13 (10.0%) patients, respectively.

Procedures were conducted under general anesthesia in 80.8% of the cases. Vascular access was TF in 123 (94.6%) patients, TSC in six (4.6%), and transaortic in one (0.8%). PCI was performed concomitantly with TAVI in eight (6.2%) cases. Valve pre-dilatation was performed in 107 (82.3%) patients. CoreValve prosthesis was implanted in 132 (97.0%) patients, and Edwards-Sapien XT prosthesis in four (3.0%). The number of rapid pacing runs was 1.0 (1.0-2.0). Maneuvers to correct paraprosthetic regurgitation were conducted in 43 patients, of which 38 (36.9%) underwent post-dilatation, four (3.1%) underwent implantation of a second valve, and one (0.8%) underwent bow traction.

Mean LV-aortic gradient on TTE was reduced from 45.5 (34.0-57.3) mm Hg at baseline to 7.0 (5.0-10) mm Hg (p<0.001) after the procedure. At the end, moderate paraprosthetic regurgitation was found in 7 (5.4%) patients.

Thirty (23.1%) cases required implantation of a new permanent pacemaker. There were vascular complications in seven (5.4%) patients. A total of 28 (21.5%) patients were subjected to blood transfusion: of which 10 (7.6%) received one red blood cell (RBC) unit, 9 (6.9%) received from 2 to 3 RBC units, and 9 (6.9%) received 4 RBC units or more.

Acute renal failure (ARF) was observed in 31 (23.8%) patients, of which 25 (19.5%), 4 (3.1%), and 2 (1.6%) were classified into stages I, II and III, respectively, in the first 72 hours. Hemodialysis was performed in 5 (3.9%) patients during hospitalization. Platelet count ranged from 194 (158-237) thousand/mm^3^ to 135 (101-165) thousand/mm^3^, with nadir at 72 hours (p < 0.0001).

Implantation success was obtained in 115 (88.5%) patients. Length of hospital stay after TAVI was 7 (6-7) days, ranging from 3-212 days.

Intra-hospital mortality occurred em 8 (6.2%) patients, with 1 death after 30 days for sepsis.

### Inflammatory response before and after TAVI

SIRS was identified in 55 (42.6%) patients. Urinary or respiratory tract infections were treated with antibiotics in 13 (10.0%) patients. Blood or urine cultures were positive in 4 cases.

Leukocyte count ranged from 6675 (5535-8623) cells/mm^3^ at baseline to 10520 (8570-13800) cells/mm^3^, reaching its peak 24 hours after TAVI (p < 0.001).

Baseline CRP was 0.3 (0.2-1.0) mg/dL, and 41 (34.5%) patients showed high CRP levels (> 0.5 mg/dL). Peak CRP was 7.0 (5.3-12.1) mg/dL and occurred on the fourth day after TAVI (Figure 1).

**Figure 1:** CRP concentration in the first week. CRP: C-reactive protein.

### Follow-up at 30 days and 1 year

At 30-day follow-up, there were 7 (5.4%) deaths. Ten (7.8%) patients were readmitted, 8 of which due to CV events.

Overall 1-year mortality was 14.6%. Deaths had a CV cause in 8 (42.0%) patients, and a non-CV cause in 11 (58.0%), with a predominance of sepsis (n = 9) in the last group.

An analysis was made to compare survivors and non-survivors at 1 year (Table 2). Independent predictors of 1-year mortality were the presence of ARF, high baseline CRP, and blood transfusion ≥ 4 RBC units (Table 3), and 1-year survival curves stratified by these variables are shown in Figure 2. When we assessed patients only after hospital discharge, we observe that baseline CRP > 0.5 mg/dL remained as an independent predictor of 1-year mortality (Table 4).

**Table 2:** Characteristics of non-survivors and survivors at 1 year follow-up

Characteristics		Non-survivors n = 19	Survivors n = 111	HR (95%CI)	p-value
Age (years)		84 (81-87)	83 (80-87)	-	0,3
Male sex		36.8%	54.1%	-	0,2
BMI (kg/m2)		26.2 (22.6-27.4)	25.2 (22.5-30.1)	-	0,8
NYHA FC IV heart failure		57.9%	38.7%	-	0,1
Diabetes mellitus		42.1%	36.0%	-	0,6
CAD		42.1%	36%	-	0,6
PVD		26.3%	23.4%	-	0,4
COPD		15.8%	8.1%	-	0,3
STS score (%)		17.9 (8.1-30.2)	8.1 (4.7-17.1)	1,03 (1,01-1,06)	0,02
Baseline creatinine (mg/dL)		1.3 (0.8-1.5)	1.1 (0.9-1.3)	-	0,8
LVEF (%)		55 (31-73)	64 (50.5-73.0)	0,98 (0,95-1,00)	0,04
Baseline hemoglobin (mg/dL)		11.2 (10.2-12.9)	12.0 (10.6-13.3)	-	0,4
Nadir hemoglobin (mg/dL)		8.1 (7.4-9.9)	9.8 (8.4-10.9)	0,68 (0,49-0,94)	0,01
Baseline CRP (mg/dL)		1.5 (0.2-2.8)	0,3 (0,2-0,9)	1,19 (1,06-1,34)	<0,0001
Baseline CRP > 0,5 mg/dL		68.8%	29.1%	4,70 (1,63-13,5)	0,004
Peak CRP (mg/dL)		14.3 (6.0-16.2)	7.8 (5.1-11.1)	1,14 (1,06-1,22)	<0,0001
SIRS		47.3%	41.8%	-	0,6
Post-TAVI aortic failure ≥ +2/4		5.3%	5.4%	-	0,99
Major vascular complication		10.5%	4.5%	-	0,2
Bleeding	Major	26.3%	20.7%	-	0,2
	Life-threatening	26.3%	4.5%	7,85 (2,62-23,5)	<0,001
Blood transfusion	2 to 3 RBC units	15.8%	5.5%	4,3 (1,20-15,5)	0,02
	≥ 4 RBC units	26.3%	3.6%	9,4 (3,24-27,2)	<0,001
	Stage I	52.6%	58.2%	8,2 (3,0-23)	<0,001
ARF	Stage II	10.5%	1.8%	14,4 (2,9-72)	0,001
	Stage III	5.3	0.9	14,7 (1,8-123)	0,013
New pacemaker		42.1%	19.8%	2,72 (1.09-6,8)	0,03
NYHA FC III heart failure at 30 days		38.5%	0.0%	66,8 (16-279)	<0,001

HR: hazard ratio; CI: confidence interval; BMI: body mass index; NYHA: New York Heart Association; FC: functional class; CAD: coronary artery disease; PVD: peripheral vascular disease; COPD: chronic obstructive pulmonary disease; STS: Society of Thoracic Surgeons; LVEF: left ventricle ejection fraction; CRP: high sensitive C-reactive protein; SIRS: systemic inflammatory response syndrome; TAVI: transcatheter aortic valve implantation; RBC: red blood cell; ARF: acute renal failure. The Mann-Whitney test (numeric variables) and the chi-square or Fisher exact tests (categorical variables) were used.

**Table 3:** Cox regression multivariate analysis of 1-year mortality

Variables in the model	Coefficient	SE Coef	HR	95%CI	p-value
ARF	1,983	0,624	7,43	2,1-24-7	0,001
Baseline CRP > 0.5 mg/dL	1,422	0,577	4,15	1,3-12,9	0,01
Blood transfusion ≥ 4 RBC units	1,543	0,649	4,68	1,3-16,7	0,02

SE Coef: standard error of the coefficient; HR: hazard ratio; CI: confidence interval; ARF: acute renal failure; CRP–C: reactive protein; RBC: red blood cell. Method of variable selection: stepwise forward.

**Figure 2:** Rates of 1-year survival stratified by (A) presence of ARF, (B) baseline CRP > 0.5 mg/dL, and (C) blood transfusion > 1 RBC unit. CRP – C-reactive protein; ARF: acute renal failure; RBC: red blood cell.

**Table 4:** Cox regression multivariate analysis for 1-year mortality in hospital-discharged patients

Variables in the model	Coefficient	SE Coef	HR	95%CI	p-value
NYHA FC III heart failure at 30 days	3.3	1.0	27.5	3.8-199	0.001
Male sex	-4.1	1.3	0.02	0.001-0.23	0.002
New pacemaker	2.3	0.8	10.2	2.0-52.3	0.005
Baseline CRP > 0.5 mg/dL	2.1	0.8	8.9	1.6-48.0	0.01

SE Coef: standard error of the coefficient; HR: hazard ratio; CI: confidence interval; NYHA: New York Heart Association; FC: functional class; CRP–C: reactive protein; Method of variable selection: stepwise forward.

The comparison between the groups with high (> 0.5 mg/dL) and normal baseline CPR is presented in Table 5.

**Table 5:** Characteristics of groups with baseline CRP > 0.5 mg/dL and ≤ 0.5 g/dL

Characteristics		Baseline CRP > 0.5 mg/dL n = 46	Baseline CRP ≤ 0.5 mg/dL n = 84	p-value
Age (years)		84 (80-88)	83 (80-87)	0.3
Male sex		53.7%	48.7%	0.6
BMI (kg/m^2^)		25.5 (23.3-27.2)	25.3 (22.2-30.1)	0.7
NYHA FC IV heart failure		61.0%	29.5%	0.001
Diabetes mellitus		31.7%	38.5%	0.5
CAD		58.5%	51.3%	0.4
PVD		26.8%	23.1%	0.6
COPD		14.6%	5.1%	0.09
STS score (%)		18.8 (7.7-26.6)	6.9 (4.2-15.3)	0.001
LVEF (%)		60 (44-68)	66 (52-74)	0.3
Baseline creatinine (mg/dL)		1.3 (0.9-1.5)	1.1 (0.9-1,3)	0.06
Baseline hemoglobin (mg/dL)		11.8 (10.0-13.2)	12.1 (10,9-13.3)	0.2
Nadir hemoglobin (mg/dL)		9.3 (8.0-10.9)	9.9 (8.4-10.6)	0.5
Baseline platelets (x10^3^/mm^3^)		200 (145- 287)	194 (165-226)	0.4
Nadir platelets (x10^3^/mm^3^)		149 (101-192)	125 (102-152)	0.04
Peak CRP (mg/dL)		11.5 (6.5-14.8)	7.2 (4.6-10.3)	0.002
Aortic failure after TAVI ≥ +2/4		2.4%	6.4%	0.6
Bleeding	Major	14.6%	25.6%	0.2
	Life-threatening	9.8%	6.4%	
Blood transfusion	2 to 3 RBC units	9.8%	3.8%	0.4
	≥ 4 RBC units	7.3%	6.4%	
	No ARF	66.7%	80.8%	0.04
ARF	Stage I	25.6%	17.9%	
	Stage II	7.7%	0%	
	Stage III	0%	1.3%	
SIRS		47.5%	42.3%	0.6
New pacemaker		26.8%	21.8%	0.6

hs-CRP: high sensitive C-reactive protein; BMI: body mass index; NYHA: New York Heart Association; FC: functional class; CAD: coronary artery disease; PVD: peripheral vascular disease; COPD: chronic obstructive pulmonary disease; STS: Society of Thoracic Surgeons; LVEF: left ventricle ejection fraction; TAVI: transcatheter aortic valve implantation; RBC: red blood cell; ARF: acute renal failure; SIRS: systemic inflammatory response syndrome. The Mann-Whitney test (numeric variables) and the chi-square or Fisher exact tests (categorical variables) were used.

Peak CRP ≥ 10.0 mg/dL had a sensitivity of 64.7% and specificity of 69.2% for 1-year mortality on the receiver operating characteristic curve, with area under the curve = 0.71 (95% confidence interval [CI], 0.57-0.86; p = 0.005). Peak CRP after TAVI was a predictor of 1-year mortality only in the univariate analysis, with hazard ratio (HR) = 1.14 (95%CI, 1.06-1.22; p < 0.0001).

## Discussion

This study assessed the impact of inflammatory response on 1-year mortality after TAVI through CRP levels in the pre- and post-operative periods, with predominance of CoreValve placement via TF access. Low intensity chronic inflammation (CRP > 0.5 mg/dL) before TAVI occurred in one third of the patients and was an independent predictor of 1-year mortality (HR 4.1; p = 0.01). Peak CRP was observed from the third to the forth days, with peak CRP ≥ 10 mg/dL being associated with greater mortality, but this was influenced by the presence of ARF and large blood transfusions.

The assessment of prognosis through inflammatory biomarkers before-TAVI was also performed by Sinning et al.,^7^ who reported that the inflammatory biomarker GDF-15 and the surgical risk score EuroSCORE II were the best predictors of 1-year mortality after TAVI. In their study, pre-TAVI CRP led to higher risk of mortality (HR 1.2; 95%CI 1.0-1.4; p = 0.012). Similarly, we found that median pre-TAVI CRP indicated higher risk of 1-year mortality (HR 1.2; 95%CI 1.0-1.3; p < 0.001). However, we believe that analysis of CRP as a categorical variable showed to be more useful, especially when adopting the cutoff value of > 0.5 mg/dL, based on publications that involved heart surgery^8^ and, more recently, TAVI.^9,10^

High CRP in the preoperative period of heart operations was associated with higher mortality in the study by Cappabianca et al.,^8^ who assessed preoperative CRP among 597 patients subjected to different types of heart surgery (SAVR in 15%) and observed that those with CRP > 0.5 mg/dL evolved to higher mortality at 3-years follow-up (odds ratio [OR], 1.93; p = 0.05). Reference values for CRP < 0.3 mg/dL were proposed based on an epidemiological study that assessed CV events without performing invasive procedures and may not represent the best cutoff value in the surgical context.

In this study, high baseline CRP was associated with decompensated HF, with greater proportion of patients in functional class IV and higher levels of brain natriuretic peptide (BNP). Villacorta et al.^11^ showed that patients with LV systolic dysfunction and decompensated HF showed higher CRP levels on admission. Jensen et al. described the relationship between CRP and BNP in decompensated HF.^12^ However, it is little likely that the worse prognosis related to CRP levels may be exclusively attributable to its relationship with HF, since more than a half of death had a non-CV cause.

Chronically high CRP has also been described among older adult, with growing evidence that chronic systemic inflammation has an impact on quality of life and on survival. The expression inflammaging was proposed to describe the many conditions related to the presence of inflammation in older adults.^13^ A meta-analysis identified 20 circulating blood biomarkers that could be potentially used in the prognostic assessment of older adults, with CRP being a predictor of overall mortality (HR = 1.4; p < 0.001) and CV mortality (HR = 1.3; p = 0,03).^14^ In the present study, no relationship was observed between CRP and advanced age, but higher STS scores were observed in the group with high baseline CRP (19% vs 7%; p = 0,001), which suggests that it may be correlated with patients’ overall health. The group with pre-TAVI CRP > 0.5 mg/dL had intra-hospital outcomes with higher peak CRP and ARF, in addition to more severe thrombocytopenia.

After TAVI, CRP kinetics in response to the procedure during the first week reached its peak between 72-96 hours, with high values up to the seventh day, in line with other studies.^9,15^ CRP kinetics in patients subjected to TAVI via TF is different from that found in SAVR.

Traditionally, the peak value of an inflammatory biomarker is considered the maximal inflammatory response obtained. In the short term, peak CRP was assessed by Krumsdorf et al.^9^, who observed, in an univariate analysis, that CRP ≥ 10 mg/dL was associated with higher 30-day mortality. This short-term finding was not confirmed by Ruparelia et al.^15^ In the long term, the prognostic value of high CRP values has not been described yet. In the present study, it was found that peak CRP ≥ 10mg/dL was able to predict 1-year mortality (HR = 3.74; p = 0.009); however, this variable was not an independent factor.

In the present sample, no association was found between some technical aspects and degree of inflammation, such as number of rapid pacing runs or direct implantation (without balloon pre-dilatation before TAVI). Sinning et al.^16^ observed a correlation between SIRS e o number of rapid pacing runs and/or post-dilatation. Ruparelia et al.^15^ found a higher peak CRP on the third day among patients who underwent pre-dilatation (11.0 [0.8] mg/dL vs 5.1 [0.3] mg/dL; p < 0.001).

In the present study, independent post-operative predictors of poor prognosis at 1 year were ARF and large blood transfusion (≥ 4 RBC units), confirmed by other authors.^17,18^ CV deaths (42%) were almost as frequent as non-CV deaths (58%), which was consistent with the PARTNER trial.^19^

The assessment of prognostic factors related to TAVI has several implications that relate from surgical strategy to assessment of procedure futility. The contribution of this study may aid other studies in the comparison of techniques and valve prostheses. It is important to emphasize that the prostheses used in this study were soon replaced with new versions that require smaller introducer sheaths, which may reduce vascular complications. Therefore, this study may be used as a parameter for future comparisons.

The present study had limitations related to its observational, retrospective and non-consecutive design. Although its sample represents one of the largest unicentric national investigations, sample size was small compared with that of multicentric international studies, and the CV events were not assessed in an event adjudication center. Levels of CRP and BNP at post-discharge follow-up could clarify the relationship between HF and valve inflammation, as well as the potential inflammatory role of unresected valve leaflets that remained incarcerated by the implanted valve prosthesis.

## Conclusions

Pre-TAVI CRP > 0.5 mg/dL is present in one third of the cases and showed to be an independent predictor of 1-year mortality, as well as the presence of ARF and large blood transfusions. Peak CRP occurs from the third to the fourth day after TAVI and, when reaching ≥ 10 mg/dL, it is correlated with higher 1-year mortality, although being dependent on other factors, such as ARF and blood transfusion.
